# Reprocessing of single-use energy devices: efficacy cleaning aspect

**DOI:** 10.1017/ash.2025.78

**Published:** 2025-09-03

**Authors:** Cham Vu, Yen Nguyen, Thoa Trinh, Huong Lu, Van Thai, Tam Duong, Duy Nguyen, Huyen Nguyen, Thanh Tran, Tran Nguyen, Thuy Pham, Tuan Huynh

**Affiliations:** 1 University Medical Center, HCM City, Viet Nam; 2 University Medicine and Pharmacy at HCM City, Viet Nam

## Abstract

**Objectives:** Energy devices (EDs), such as Harmonic, Ligasure, Thunderbeat and Trocar are widely used in Minimally Invasive Surgeries. They are expensive and designed for single use. However, due to the limitation of resources, they have been reused in some cases. Therefore, we aimed to assess the efficacy of EDs reprocessing by Adenosine Triphosphate (ATP) method. **Methods:** This was a cross-sectional description study. After first clinical using, EDs were taken to cleaning. Every ED was cleaned three times. Efficacy cleaning was assessed after each cleaning procedure by ATP method. ATP <200 RLUs (Relative Light Units) was benchmark as efficient cleaning process. **Results:** A total of 611 EDs were studied, including 269 of Harmonic, Ligasure, Thunderbeat, 298 of Trocar, and 44 other types. Detachable devices accounted for about 32.7%. Overall, after three consecutive cleanings, the median ATP values were decreased dramatically (957 RLUs, 160 RLUs, and 62 RLUs, respectively). This was a significant reduction in ATP levels between three stages (p < 0.05). There were 63.5%, 84.3%, and 92.8% EDs that had ATP < 200 RLUs after first, second, third cleaning respectively. Approximately 90% of EDs were still functional after three cleaning times. Nondetachable items were to be more difficult to clean than detachable ones (p = 0.0003, OR 1.3 [1.1 – 1.5]). **Conclusions:** Our data suggest that monitoring efficacy cleaning of surgical instruments in general and single-use energy devices in particular with ATP can identify a number of different influence factors, like the instrument condition, reprocessing procedure, or especially their structure. ATP measurement seems to be a valid technique that allows an immediate repeat of the manual cleaning if the results exceed the established cutoff of 200 RLUs.

